# An Investigation of Fibulin-2 in Hypertrophic Cardiomyopathy

**DOI:** 10.3390/ijms21197176

**Published:** 2020-09-29

**Authors:** Ayman M. Ibrahim, Mohamed Roshdy, Sara Elshorbagy, Mohammed Hosny, Sarah Halawa, Dina Yehia, Hasnaa A. Elfawy, Ahmed Eldessouki, Faisal Mohamed, Amany Ellithy, Mohamed Abdelfattah, Amr Elsawy, Mohamed Elkhatib, Mona Allouba, Ahmed Elguindy, Yasmine Aguib, Magdi Yacoub

**Affiliations:** 1 Aswan Heart Center, Aswan 200, Egypt; mohamed.roshdy@aswanheartcentre.com (M.R.); sara.shorbagy@aswanheartcentre.com (S.E.); dr.mohammedhosny8530@gmail.com (M.H.); s_halawa@aucegypt.edu (S.H.); dina.yabbas@gmail.com (D.Y.); hasnaahmed2322@gmail.com (H.A.E.); ahmad.disk@hotmail.com (A.E.); fiesal.mohamed@aswanheartcentre.com (F.M.); amanyellithy@gmail.com (A.E.); m.fattah.youssef24@gmail.com (M.A.); amr.elsawy@aswanheartcentre.com (A.E.); elkhateb60@gmail.com (M.E.); monaallouba@gmail.com (M.A.); ahmed.elguindy@aswanheartcentre.com (A.E.); 2 Department of Zoology, Faculty of Science, Cairo University, Giza 12613, Egypt; 3 Cardiology Department, Faculty of Medicine, Cairo University, Giza 11562, Egypt; 4 Biotechnology Graduate Program, American University in Cairo, New Cairo 11835, Egypt; 5 National Heart and Lung Institute, Imperial College London, London SW3 6LY, UK; 6 Heart Science Centre, Harefield Hospital, Uxbridge UB9 6JH, UK

**Keywords:** extracellular matrix, cardiac remodeling, fibrosis, hypertrophy, fibroblast

## Abstract

Hypertrophic cardiomyopathy (HCM) is the most common inherited heart muscle disease, with a prevalence of at least 1 in 500 in the general population. The disease is pleiotropic and is characterized by an increased stiffness of the myocardium, partly due to changes in the extracellular matrix (ECM), with elevated levels of interstitial fibrosis. Myocardial fibrosis is linked to impaired diastolic function and possibly phenotypic heterogeneity of HCM. The ECM consists of a very large number of proteins, which actively interact with each other as well as with myocardial cells. The role of other multiple components of the ECM in HCM has not been defined. Fibulin-2 is a glycoprotein component of the ECM, which plays an important role during embryogenesis of the heart; however, its role in adult myocardium has not been adequately studied. We here describe, for the first time, abnormal expression of fibulin-2 in the myocardium in patients with HCM as compared to normal controls. This abnormal expression was localized in the cytoplasm of myocardial cells and in the interstitial fibroblasts. In addition, fibulin-2 levels, measured by ELISA, were significantly elevated in the serum of patients with HCM as compared to normal controls.

## 1. Introduction

Hypertrophic cardiomyopathy (HCM) is the most common inherited heart muscle disease, with a prevalence of at least 1 in 500 in the general population. The disease is pleiotropic and is characterized by structural and functional changes of the different components of the myocardium, including cardiac myocytes and extracellular matrix [1,2]. The extracellular matrix (ECM) plays an important part in the pathophysiology of HCM [2,3]. It consists of a very large number of proteins, which actively interact with each other as well as myocardial cells [4,5,6]. The exact role of the different components of the ECM in HCM has not been defined.

Fibulins (FBLNs) are a family of ECM glycoproteins with different physiological functions [7,8,9]. FBLNs 1 and 2 are the largest proteins in the family and are known for their interchangeable interaction with other ECM proteins, such as collagens, tropoelastin, and perlecans [10,11]. In cardiac tissue, FBLNs 1 and 2 are overexpressed in the migratory mesenchymal cells during the development of mouse valvular septum [10,11,12]. They are also expressed in different types of blood vessels along with other FBLNs [13]. FBLN2 was originally identified in the embryonic endocardial cushion tissue and the heart valves [10]. It has been associated with the development and remodeling of tissues, as it is expressed at sites of epithelial-mesenchymal transition during endocardial formation in the developing heart and during neural crest development [14]. The role of FBLN2 in HCM and cardiac ECM remodeling is not studied.

We here describe the abnormal expression of FBLN2 in the myocardium and importantly elevated levels in the serum of patients with HCM, giving an initial insight into the clinical relevance of these findings.

## 2. Results

### 2.1. Cohort Characteristics

In all, 132 subjects were clinically examined (total of 95 HCM and 37 ‘normal’ controls). The HCM patients had evidence of significant LV outflow obstruction requiring myectomy [15]. Control serum samples were used from the Ecco-Gen EHVol study: (Accession no: EGAS00001004434) who were fully phenotyped with respect to cardiovascular health [16].

Detailed experimental design and samples origin are described in the methods section. The demographics of HCM patients and normal controls are shown in Table 1.

### 2.2. FBLN2 Is Upregulated in the Myocardial Tissue and Is Localized to the ECM and Cardiomyocytes

**Figure 1:**
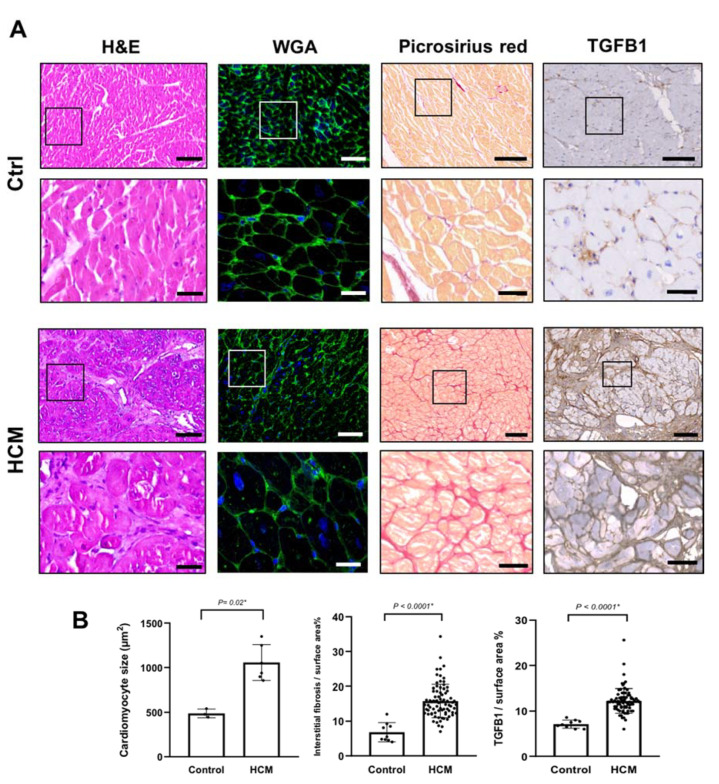
Histological characterization of HCM myectomy tissues (**A**) Histological characterization of HCM tissues compared to control tissues using hematoxylin and eosin (H&E) staining, WGA staining, Picro-sirius staining, and TGFβ1 expression, assessed by immunohistochemistry. Scale bars are 100 (overview) and 20 µm (magnified insets). (**B**) Bar plots show a significant increase of cardiomyocytes size (µm^2^) (6 HCM vs. 3 ctrls), interstitial fibrosis index (79 HCM vs. 9 ctrls), and TGFβ1 levels (79 HCM vs. 9 ctrls).

To assess the general cellular and histological changes, and the level of interstitial fibrosis in the HCM samples, we performed hematoxylin/eosin (H&E) and picro-sirius red staining, in addition to Transforming growth factor beta (TGFβ1) immunohistochemistry on myocardial specimens from HCM patients (*n* = 79) and compared them to control tissues (*n* = 9). Wheat germ agglutinin (WGA) staining was performed in six representative HCM tissues compared to three controls. HCM myocytes were significantly larger in size by (2-fold) (*P* = 0.02) and tissues showed a characteristic myocardial disarray and a significantly higher level (3-fold) of interstitial fibrosis in contrast to control tissues (*P* < 0.0001 *) (Figure 1). Immunohistochemical analysis also showed a significant increase in the level of TGFβ1 expression in HCM tissues (2–3-fold) (*P* < 0.0001 *), localized to the areas where interstitial fibrosis was visible (Figure 1).

**Figure 2:**
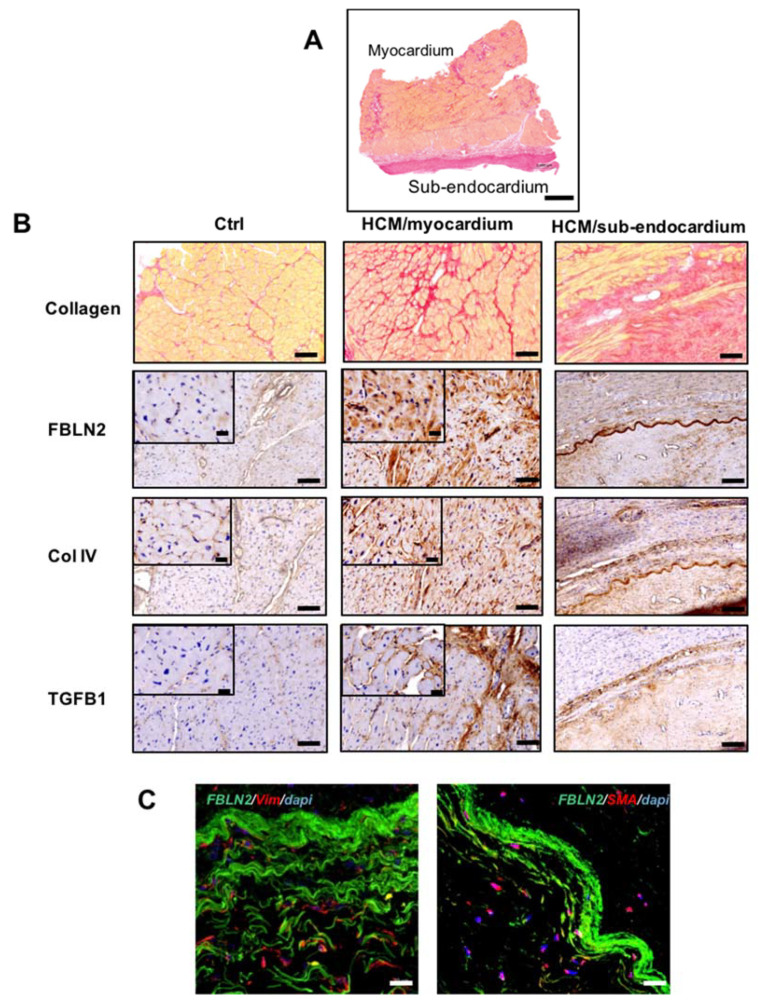
FBLN2 expression and localization in HCM myectomy tissues along with Col IV and TGFβ1. (**A**) A representative transmural section of a myectomy specimen stained with picro-Sirius red and shows fibrosis distribution in the myocardium and sub-endocardium. Scale bar is 1 mm. (**B**) Picro-sirius red and immunohistochemical analysis of HCM tissues at the myocardium and sub-endocardial (*n* = 79), compared to controls (*n* = 9), showing FBLN2, Col IV, and TGFβ1 expression. Scale bars are 100 (overview) and 20 µm (magnified insets). (**C**) Confocal microscopy imaging shows Vim+ and SMA+ cells (red) infiltrating the FBLN2 sheath (green) at the sub-endocardial region (representative of 3 HCMs vs. 3 controls). Scale bars are 20 µm.

Next, we aimed to investigate the expression pattern of FBLN2 in myectomy specimens in contrast to control tissues, and we also assessed its expression along with tissue remodeling markers, TGFβ1 and Collagen IV (Col IV). Immunohistochemical analysis of FBLN2 in control myocardial tissues showed very low expression, with a slight increase around micro-vessels (Figure 2B). In contrast, HCM tissue sections showed a much higher expression of FBLN2 localized in myocytes cytoplasm and cardiac stroma. However, some areas of HCM myocardial tissues showed an FBLN2 expression level similar to controls (Figure 2B and Supplementary Material Figure S1). Further, myocardial regions exhibited an elevated expression of both TGFβ1 and Col IV, which were both mainly localized to the interstitial stroma.

In addition, FBLN2 was also localized to the sub-endocardial tissue in the form of a dense sheath, where fibrous collagen is condensed (Figure 2B). This expression co-localized with Col IV and TGFβ1 expressions, and this was evident via consecutive tissue staining for the three markers (Figure 2B). To further characterize the microstructure of the observed FBLN2-rich sheath at the sub-endocardial region, we employed immunofluorescence and confocal microscopy analysis to specifically assess whether fibroblasts and/or myofibroblasts are associated with this area, and we identified vimentin (Vim)+ and smooth muscle actin (SMA)+ cells infiltrating the FBLN2 sheath.

### 2.3. Total Expression of FBLN2 Is Elevated in HCM Myocardium

**Figure 3:**
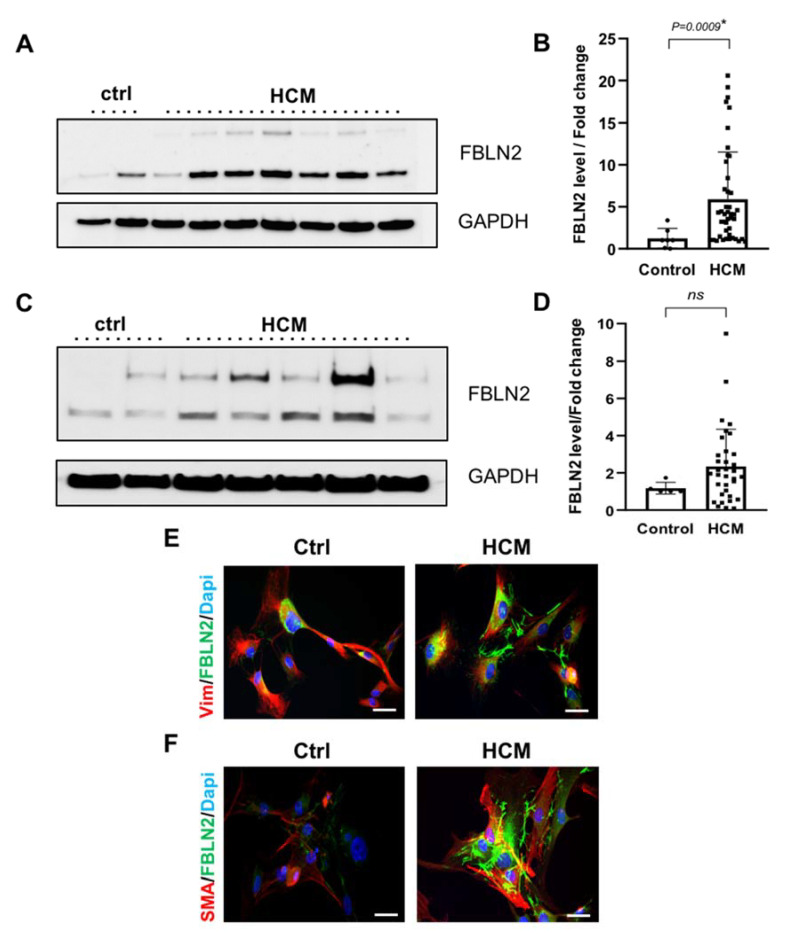
Elevated expression of FBLN2 in HCM myectomy tissues and cultured fibroblasts. (**A**) A representative Immunoblot for HCM and ctrl tissues shows total FBLN2 protein expression normalized to Glyceraldehyde 3-phosphate dehydrogenase (GAPDH) expression. (**B**) Bar plot shows a significant difference (*P* = 0.0009) between mean FBLN2 expression in HCM tissues (*n* = 44) compared to ctrl tissues (*n* = 7). (**C**) A representative Immunoblot for cultured HCM and ctrl fibroblasts shows total FBLN2 protein expression normalized to GAPDH expression. (**D**) Bar plot shows an elevated level of FBLN2 in cultured HCM fibroblasts (*n* = 34) compared to ctrl fibroblasts (*n* = 5). E and F: Confocal microscope imaging of cultured HCM (*n* = 3) and control fibroblasts (*n* = 3), shows FBLN2 expression pattern and localization. Cells were co-stained with FBLN2 and vimentin (mesenchymal fibroblasts marker) (**E**), and FBLN2 with smooth muscle actin (myofibroblasts marker) (**F**). Scale bars are 20 µm.

To validate the histological analysis and to assess the overall FBLN2 tissue expression levels, total protein lysates from HCM myectomy tissues and cultured fibroblasts were analyzed for FBLN2 expression using immunoblotting. Quantification of FBLN2 protein expression in HCM myocardial lysates (*n* = 44) showed significantly higher levels of FBLN2 compared to control tissues (*n* = 7) (*P* = 0.0009) (Figure 3A,B and Supplementary Material Figure S3).

Expression of FBLN2 in HCM cultured fibroblasts (*n* = 34) showed a tendency to be higher, which did not reach statistical significance, compared to fibroblasts from controls (*n* = 5) (Figure 3C,D and Supplementary Material Figure S4).

Further, immunofluorescence and confocal imaging analysis of three representative cultured fibroblasts derived from HCM patients showed elevated FBLN2 levels, compared to control fibroblasts. The protein was localized to the cell matrix. Cells were co-stained with FBLN2 and fibroblasts markers (vimentin and SMA) to confirm cells’ purity (Figure 3E,F).

To further examine the significance of these findings, we next assessed the amount of circulating FBLN2 in patients’ and controls’ serum.

### 2.4. HCM Is Associated with High Levels of Circulating FBLN2

**Figure 4:**
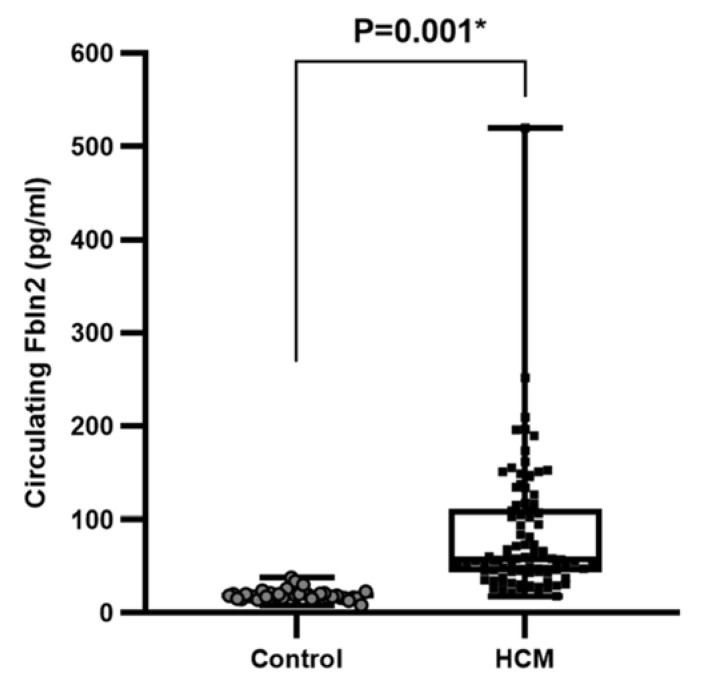
Increased FBLN2 expression in the serum HCM patients. A boxplot shows a significant increase in circulating FBLN2 (pg/mL) in HCM patients’ serum (*n* = 90) compared to healthy subjects (*n* = 37).

To assess whether FBLN2-elevated tissue expression is reflected in patients’ circulation, we next employed ELISA to measure circulating FBLN2 in HCM patients (*n* = 90) and compared them to serum samples from healthy volunteers (*n* = 37). The clinical characterization/phenotyping of healthy controls cohort are exhibited in Table 1. Serum levels of FBLN2 were significantly higher in HCM group in contrast to the healthy controls group (mean 92.77839 ± 61.1 pg/mL compared to 20.004 ± 5.83 pg/mL, *P* < 0.001) (Figure 4).

## 3. Discussion

This study shows, for the first time, increased levels of FBLN2 in the myocardium and serum of patients with hypertrophic cardiomyopathy. The abnormal expression in the myocardium was localized mainly to the extracellular matrix. These findings suggest an important role of FBLN2 in the pathophysiology of HCM. Further investigation is required to translate FBLN2 expression in the cardiac tissue microenvironment and in blood circulation to HCM-associated cardiac dysfunction.

The ECM is a complex structure, which can play a role in the pathobiology of HCM, both during morphogenesis, and in the established disease [1,2] where it can influence phenotype [17]. The exact mechanisms of these intricate interactions at molecular and cellular levels is the subject of extensive research [1,3,4,18]. Various cardiac components can contribute to the disease development, which can start very early at embryonic and postnatal cardiac development [17]. At the structural level, the ECM in the form of fibrosis is markedly increased, around the cells, in septa, scars, as well as in the walls of intramyocardial blood vessels (Figure 1 and Figure 2) [17,19,20]. Apart from affecting the physical properties of the myocardium, there is cross talk between molecular components of the ECM and myocardial cells [19], which have important functional implications. This cross talk occurs via binding of myocardial cells’ integrins, in addition to cytoskeletal proteins, to various ECM proteins in the cardiac tissue microenvironment [20]. The accurate representation of fibrosis in HCM patients with various disease stages is the subject of several recent studies, either by cardiac magnetic resonance (CMR) alone or combined with histological examination [3,21].

FBLN2 expression is reported in the embryonic cardiac cushion that develops into heart septa and contributes to valvular formation [9,10]. Studies have investigated the expression pattern and the function of FBLN2 in the context of ECM remodeling and functional morphogenesis, considering its capability to interact with various ECM proteins, such as versican, perlican, and nidogen [8,9,22]. FBLN2 has been identified in myoepithelial cells during mammary morphogenesis associated with ECM remodeling and basement membrane restructuring around the epithelium [22]. During vascular development, FBLN2 binds to tropoelastin and thus serves as an interface between the elastin core and fibrillin microfibrils [7].

Few studies have investigated FBLN2 function during mouse heart formation and induced hypertrophy [12,23,24]. These studies have suggested that FBLN2 is important for mediating angiotensin II-dependent TGFβ1 activation, which facilitates the fibrosis cascade in hypertrophied mouse hearts. During cardiovascular disease, FBLN2 has also been identified in the wall of the aorta, contributing to the ECM pool and mediating changes in the aortic wall structure upon injuries, such as thoracic aortic aneurysm [25].

In this study, FBLN2 expression was evidently elevated in the myocardium and was clearly localized at the sub-endocardial fibrotic tissue excised from patients with obstructive HCM. FBLN2 has recently been shown to interact with COL IV to stabilize the basement membrane [26]. At the sub-endocardial region, FBLN2 co-localized with Col IV and TGFβ1. One possible explanation to this finding is that FBLN2 interacts with the BM during remodeling in this active traumatic region. High col IV expression has also been associated with high levels of fibrous collagen deposition in hepatic fibrosis lesions at the sites of perisinusoidal BM formation [27], which is proposed to be similar to the scarring formation at the studied sub-endocardial tissue. Further, FBLN2 and TGFβ1 co-expression, especially in high fibrotic regions, is consistent with the hypothesis that FBLN2 is required for TGFβ1 activation and interstitial fibrosis [23,28]. Loss of FBLN2 has been suggested to attenuate angiotensin-II signaling via a reduction of TGFβ1 activation [23] and can further protect against ventricular dysfunction in FBLN2-null mice after myocardial infarction [29]. FBLN2 mediation in TFGβ1 activation and ECM remodeling should be functionally investigated and the exact mechanism of FBLN2 with TFGβ1, Col IV, and other relevant players requires further studies.

As fibroblasts are key players in ECM remodeling [4,6,18], we hypothesized that fibroblasts contribute to FBLN2 secretion and in accordance, we identified vimentin+ and SMA+ cells at the sub-endocardial region with FBLN2 expression, and we reported elevated levels of FBLN2 in HCM-associated fibroblasts in a subset of HCM patients. The notable heterogeneity among patients’ fibroblast lines is intriguing and can be explained by reported HCM pleiotropy and phenotype heterogeneity [17,19,20,30]. Because this was a retrospective study, we were not able to correlate FBLN2 levels in tissues and their corresponding associated cells because, according to unshown data, this requires sampling of the fibroblasts from multiple trackable tissue regions. This and the study of proteomes and transcriptomes will be the subject of a future investigation [31].

FBLN2 is a secreted soluble ECM protein [14]. To our knowledge, the level of FBLN2 in the circulation of HCM patients has not been studied to date. Based on FBLN2 expression at the sub-endocardial tissue, very close to the LV lumen, we hypothesized that it can be shed in the blood and be detected in the circulation. High levels of FBLN2 in the sera of HCM patients was consistent with its elevated levels in the tissue. Correlations between clinical parameters and FBLN2 expression with an adequately powered sample size will be studied in the future.

In conclusion, we introduce FBLN2 as a matrix protein contributing to the extracellular matrix changes in cardiac tissue in obstructive HCM and sheds light on FBLN2 as a possible circulating marker of (cardiac) fibrosis. This work proposes future studies to further dissect the underlying mechanisms and the clinical impact of these findings.

## 4. Materials and Methods

### 4.1. Ethics, Study Cohort, Sample Collection, and Experimental Design

All subjects from HCM and healthy control cohorts gave their informed consent for inclusion before they participated in the study. The study was conducted in accordance with the Declaration of Helsinki, and the protocol was approved by the local Research Ethics Committee (Hypertrophic cardiomyopathy Project identification code: 20130405MYFAHC_CMR and REC number: 20130405MYFAHC_CMR_20130330/approval date 09 June 2015; Healthy Volunteer project [16] identification code: 20151125MYFAHC_Hvol and REC number: 20151125MYFAHC_Hvol_20161027/approval date 27 October 2016).

This retrospective study included 95 consecutive obstructive HCM patients who were eligible for septal myectomy surgery. Clinical evaluation of symptoms and signs of these HCM patients was done in a special outpatient clinic in AHC. Transthoracic echocardiography with the recommended views, and relevant measurement, in addition to cardiac CMR with the recommended sequences, were done for all patients according to the ESC guidelines for management of HCM patients [32]. In addition, the study included 37 control serum samples from the Ecco-Gen EHVol study (Accession no: EGAS00001004434) [16]. These individuals are fully phenotyped with respect to cardiovascular health.

Blood samples were collected from pre-operative patients and processed for serum isolation within 30 min from the time of collection. Serum aliquots were stored at −80 °C at the AHC Biobank. Tissue samples excised from the obstructive region of the left ventricle (LV) were collected upon surgery; myectomy specimens were divided into symmetrical portions and collected in 10% neutral buffered formalin (NBF) (Sigma, St. Louis, MO, USA, #HT501128) for histological analysis, complete media (DMEM-F12-10% FBS) (Gibco^TM^, Waltham, MA, USA, #31331028) (fetal bovine serum/FBS (GibcoTM, #10082147) for immediate fibroblast isolation, and flash frozen in liquid nitrogen, and stored in −80 °C for RNA and protein analysis.

Control cardiac septal fibroblasts and tissue were obtained from Magdi Yacoub institute (MYI), UK (based on a material transfer agreement); one commercially available Human Ventricular Cardiac Fibroblasts (Lonza, Basel, Switzerland, NHCF-V, #CC-2904) was used.

Histological examination in tissues was performed on 79 HCM vs. 9 controls. FBLN2 quantification with immunoblotting in myocardial tissues was performed on 44 HCM vs. 7 controls. FBLN2 quantification with immunoblotting in cultured fibroblasts was performed on 34 HCM vs. 5 controls. Circulating FBLN2 was assessed in 90 HCM vs. 37 controls from the Ecco-Gen EHVol study [16].

### 4.2. Histological Analysis

Fixed tissue was processed using an autoprocessor machine (Leica, Wetzlar, Germany) via dehydration with increasing concentrations of ethanol, followed by clearing with xylene, and then embedded in pure paraffin wax (Sigma, #327204). Then, 5-μm sections of FFPE tissue sections were deparaffinized in xylene (Sigma, #534056) for 10 min, hydrated in decreasing concentrations of alcohol (Merck, Burlington, MA, USA, #100983), and then immersed in tap water. For hematoxylin/eosin staining (H&E), sections were stained in hematoxylin (Dako, Glostrup, Denmark, #S3309) for 30 s, washed with tap water (alkaline medium), and then stained with eosin (Dako, #CS701) for 1 min. For collagen I and III staining, slides were incubated in picrosirius red stain (Abcam, Cambridge, UK, #ab150681) for 1 h then washed in 0.5% acetic acid for differentiation. Stained sections were dehydrated with incremental concentrations of ethanol, cleared with two changes of xylene (Sigma, #534056), and then mounted with DPX (Sigma, #06522). Mounted sections were scanned with a slide scanner (Zeiss, Oberkochen, Germany) and analyzed using Zeiss blue software.

### 4.3. Interstitial Fibrosis Examination

Picrosirius red-stained sections were scanned using the Axioscan system (Zeiss). Images were captured including areas of cardiomyocytes/matrix interface, and excluding areas with blood vessels, usually surrounded by a dense layer of collagen. Image analysis was performed using ImageJ software (NIH) as follows: Images were converted to 16-bit format, fibrous collagen (in red) was subtracted from the yellow background, and then red stain was quantified as surface area fraction/percentage from the whole area of the picture. For each assessed section, four images were captured of different fields and the mean collagen surface area per sample was calculated, 20.

### 4.4. Wheat Germ Agglutinin (WGA)

Sections were gradually rehydrated, washed thrice with distilled water, and then washed twice with 1X PBS (Lonza, #17516F) for 5 min each. Sections were incubated with WGA stain (1:100) (Thermo Scientific™, Waltham, MA, USA, #W11261) for 45 min in a dark humidified chamber, and washed twice with 1X PBS for 5 min. Washed sections were mounted with Dapi-containing mounting media (Invitrogen™, #P36962) and stored at 4 °C. Mounted sections were scanned with a confocal microscope (Zeiss) and analyzed using Zeiss black software (Zeiss).

### 4.5. Immunohistochemistry and Immunofluorescence

First, 5-μm FFPE sections, on positively charged slides, were deparaffinized in xylene (Sigma, #534056) for 10 min, hydrated in decreasing concentrations of alcohol (Merck, #100983), and then immersed in tap water. Antigen retrieval was performed using 1 mM EDTA (Sigma, #60-00-4) buffer (pH 8) under high pressure and all other incubations were performed at RT using a humidity chamber. Sections were blocked with pre-diluted 2.5% goat serum (GibcoTM, #16210-064) for 20 min then incubated with primary antibody for 2 h. All antibodies were diluted to their final concentrations using blocking solution (FBLN2 1:200 (Thermo Scientific™, #PA5-21640), COL IV 1:200 (Abcam, #ab6586), TGFβ1 1:150 (abcam, ab170874), and SMA 1:300 (Thermo Scientific™, # MS-113-P). For colorimetric reactions, washed tissue sections were incubated for 30 min with goat polyclonal anti-rabbit (Cell signaling, #7074S)/anti mouse (HRP labelled) (Cell signaling, #7076S) secondary antibody and washed thrice prior to staining with DAB + Chromogen for 2 min. Stained tissue sections were counterstained with hematoxylin (Dako, #S3309), dehydrated through increasing concentrations of ethanol (Merck, #100983) then xylene (Sigma, #534056), before mounting with cover slips using DPX mounting medium (Sigma, #06522). For fluorescence reactions, washed tissue sections were incubated with 2ry antibodies conjugated with fluorophores, (Goat Anti-Mouse IgG H&L Alexa Fluor^®^ 594) (abcam, ab150116) and (Goat Anti-Rabbit IgG H&L Alexa Fluor^®^ 488) (Abcam, #ab150077) at a 1:500 dilution, incubated in the dark for 1 h at RT, washed thrice, and then mounted with dapi-containing mounting media (Invitrogen™, Carlsbad, CA, USA, #P36962). Stained sections were kept in the freezer for storage.

### 4.6. Fibroblasts Isolation

Myoectomy specimens were collected from the surgery into phosphate buffer saline (PBS) w/o Ca, Mg, and transported to the cell culture laboratory where the fibroblast isolation was performed immediately. Fibroblasts were isolated using the explant method of isolation. Tissues were placed on ice and cut with sterile blades into small chunks of ~2–3 mm^3^ and were allowed to stick to the T25 plate for 10 min. Tissue explants were covered with culture media consisting of: Dulbecco’s Modified Eagle Medium: Nutrient Mixture F-12 (DMEM/F-12) with GlutaMAX, 10% Fetal Bovine Serum (FBS), 1% Non-essential amino acids (Gibco^TM^, #11140050), 1% Penicillin/streptomycin (Gibco^TM^, #15140122), and 0.1% B-mercaptoethanol (Gibco^TM^, #21985023). Culture vessels were incubated at 37 °C and 5% CO_2_.

### 4.7. Western Blotting

For tissues, 0.01 g of frozen myectomy (*n* = 34) and control LV tissues (*n* = 3) were homogenized in cold RIPA buffer (Thermo Scientific™, 89900) (25 mM TRIS pH 8, 150 mM NaCl, 1% NP-40, 1% sodium deoxycholate, 0.1% SDS, and protease inhibitor) (Roche, # 4693116001).

For cells, cells were scraped off culture plates into RIPA buffer (*n* = 34), and 1-mL syringes were used to gently homogenize cells. Tissue and cell lysates were centrifuged at 4 °C for 20 min at 25,000 g to separate the soluble fraction of extracted proteins (supernatant) from cell debris (pellet). Protein lysates were quantified using a BCA™ Protein Assay Kit as per the manufacturer’s instructions (Thermo Scientific, #23227). Then, 5 μg of denatured protein per sample were electrophoresed through 10% tris gel (Bio-rad, Hercules, CA, USA, #1610173) in the presence of 1xNuPage MOPS SDS running buffer (Invitrogen™, # NP0001-02). Proteins were transferred from the gel to Whatman^®^ Protran^®^ Nitrocellulose Transfer Membrane (0.2 μm) (Thermo Scientific™, #88024) using a Biorad transfer module, protein transfer buffer (1X NuPage transfer buffer (Invitrogen™, NP00061)), 10% methanol (Merck, #1-06007-2500) in dH2O. The blot was incubated for 30 min at RT in 5% Marvel Original dried skimmed milk (Heirler, Ludwigstraße, Germany) blocking solution (5% Marvel Original dried skimmed milk in 1X PBS Tween-20 (Sigma, #P1379) wash buffer). Blot was then incubated for 2 h at RT with primary antibody (GAPDH (cell signaling, #2118S) at 1:1000, FBLN2 (Thermo Scientific™, #PA5-21640) at 1:500, and ꞵ-actin (cell signaling, #12153S) at 1:1000), washed thrice with washing buffer for 15 min, incubated for 1 h at RT with horseradish peroxidase (HRP)-labelled secondary antibody (Anti-mouse IgG, HRP-linked Antibody (cell signaling, #7076S), Anti-rabbit IgG, HRP-linked Antibody(cell signaling, #7074S), and finally washed thrice for 15 min. All of the above incubations and washes were performed using oscillating shakers. Signal development was carried out using chemiluminescence-based reaction, using the Amersham ECL Western blotting detection reagents (Thermo Scientific™, #32106) and analysis system as per the manufacturer’s instructions, and signal was detected using an Amersham Imager 600. Relative quantification of bands was performed using ImageJ software. Both FBLN2 bands (at 120 and 60 KDa) were considered in the quantification (Figure 3C,D and Supplementary Material Figures S3 and S4) [26,33].

### 4.8. Immunocytochemistry

Fibroblasts were grown in 24-well plates with fitting coverslips in each well, and let to adhere and grow till 70–80% confluency; then, cells were fixed with 4% paraformaldehyde (PFA) (Serva, #31628.01) for 20 min at RT and then incubated with chilled absolute methanol for 1 min. Cells were washed thrice with PBS, permeabilized with PBS + 0.5% Triton-100 for 10 min at 4 °C, and then rinsed with 100 mM Glycine (Sigma, #G8898) in PBS three times 10 min each. Cells were then blocked with blocking buffer (PBS + 10% FBS, 7.7 mM NaN3 (Sigma, #S2002), 0.1% BSA (Sigma, #A2153), 0.2% Triton x-100 (Sigma, #T8787), and 0.05% Tween-20 (Sigma, #P1379)) with rocking at 120 rpm for 1 h at RT, and then treated with the antibody at proper concentration diluted in blocking buffer (FBLN2 at 1:200). Cells were washed by rinsing three times (10 min each) with blocking buffer on the rocking platform and then incubated with the secondary antibody diluted in the blocking buffer, and kept in the dark for 1 h at RT. Cells were then washed with PBS three times with rocking and then mounted with mounting medium containing DAPI to counterstain the nuclei. Slides were kept at −20 °C for storage.

### 4.9. Enzyme-Linked Immunosorbent Assay (ELISA)

FBLN2 levels in 90 HCM and 37 control serum samples were assessed using a commercially available ELISA kit with monoclonal antibodies, according to the manufacturer’s instructions (Abbexa Ltd., Cambridge, UK, #abx253675). HCM serum samples were diluted 1:2. In addition, tested samples were measured in duplicates. Standards and tested samples were mixed by pipetting up and down to be homogenous before adding into the wells. The plate was then sealed and incubated in a horizontal orbital microplate shaker (500 ± 50 rpm) at 37 °C for 90 min. Prepared biotin-conjugated antibody (diluted 1:100) working solution was added into each well after discarding the previous solution without washing and incubated at 37 °C for 60 min. The solution was removed, and wells were washed thrice. Horseradish peroxidase (HRP) antibody solution (diluted 1:100) was added to the wells then incubated at 37 °C for 30 min. The solution was discarded, and wells were washed 5 times; then, 90 uL of TMB substrate were added into each well for 10–20 min. Finally, 50 uL of the sulfuric acid stop solution were added to each well to stop the reaction and then measured immediately at an absorbance of 450 nm.

### 4.10. Statistical Analysis

Continuous data are presented as median or as the mean ± standard deviation. Comparisons between the two groups of continuous measurements were performed with the Mann–Whitney test.

## Figures and Tables

**Table 1:** Clinical characteristics of hypertrophic cardiomyopathy (HCM) and normal control cohorts.

Clinical Parameters	HCM (*n* = 95)	Control (*n* = 37)
Gender, Male/Female	68/27	20/17
Age, years old	38.29 ± 13.99	25 (25–29)
Body surface area BSA. m^2^	*n* = 95 2 (1.77–2.10)	*n* = 36 1.8 ± 0.2
Body mass index	28.66 ± 5.80	24.21 (22.65–28.55)
Dyspnea	Dyspnea (*n* = 89) (93.6%) None (*n* = 6) (6.3% )	None (*n* = 37) (100%)
NYHA class	Class 1 (*n* = 10) (10.52%) Class 2 (*n* = 28) (29.47%) Class 3 (*n* = 44) (46.32%) Class 4 (*n* = 13) (13.68%)	Class 1 (*n* = 37) (100%)
Angina	Angina (*n* = 49) (51.58%) None (*n* = 46) (48.42%)	None (*n* = 37) (100%)
CSS classification	Class 1 (*n* = 46) (48.42%) Class 2 (*n* = 25) (26.32%) Class 3 (*n* = 20) (21.05%) Class 4 (*n* = 3) (3.16%)	Class 1 (*n* = 37) (100%)
Syncope	Syncope (*n* = 23) (24.21%) None (*n* = 72) (75.78%)	None (*n* = 37) (100%)
Max Septal wall thickness (cm)	*n* = 95 2.6 (2.3–3.275)	*n* = 21 0.87 (0.87–1.1)
PASP (mmHg)	*n* = 47 31 (20–39)	*n* = 11 17.54 ± 7.32
LVOTO resting gradient (mmHg)	*n* = 94 71 (50.5–96)	*n* = 37 10 ± 5
Left ventricular mass by CMR (gm)	*n* = 85 104.55 (88.44–144.47)	*n* = 25 41.33 (39.26–25.75)
Left ventricular end systolic volume by CMR (CC)	*n* = 87 18.43 (13.91–26.95)	*n* = 28 32.32 ± 8.99
Left ventricular end diastolic volume by CMR (CC)	*n* = 87 79.17 ± 20.08	*n* = 28 82.11 ± 11.58
Ejection Fraction% by CMR	*n* = 87 73.87 ± 8.87	*n* = 29 62.5 ± 8.12
Indexed Left atrial volume by CMR (mL/m2)	*n* = 85 71.7 (59.58–89.23)	*n* = 29 46.3 ± 9.45
Diastolic dysfunction (yes or no) Diastolic dysfunction Grade	Yes (33.75%) Missing data (66.31%) I (8.41%) II (17.98%) III (7.36%)	No (*n* = 37) (100%)
Cardiac Troponin I (ng/mL)	*n* = 72 0.0135 (0.008–0.0255)	*n* = 29 0.003 (0.001–0.007)

Data are presented as mean ± SD for normally distributed continuous variables, as median and interquartile range IQR for non-normally distributed continuous variables and as percentages (%) for categorical variables.

## Supplementary Materials

Supplementary Materials can be found at https://www.mdpi.com/1422-0067/21/19/7176/s1.

## Abbreviations

Ang II	Angiotensin II
BM	Basement Membrane
CMR	Cardiac magnetic resonance
ECM	extracellular matrix
FBLN	Fibulin
FFPE	Formalin-fixed paraffin embedded
HCM	Hypertrophic cardiomyopathy
H&E	Hematoxylin-Eosin
Col	Collagen
SMA	Smooth muscle actin
TGFβ	Transforming growth factor beta
Vim	Vimentin
