# Supplementary material for: An Investigation of Fibulin-2 in Hypertrophic Cardiomyopathy
Source: Int J Mol Sci. 2020 Sep 29;21(19):7176. doi: 10.3390/ijms21197176 (PMC7583916; doi:10.3390/ijms21197176)
Supplement: Supplementary file 1 [file ijms-21-07176-s001.pdf]

Supplementary Figure 1

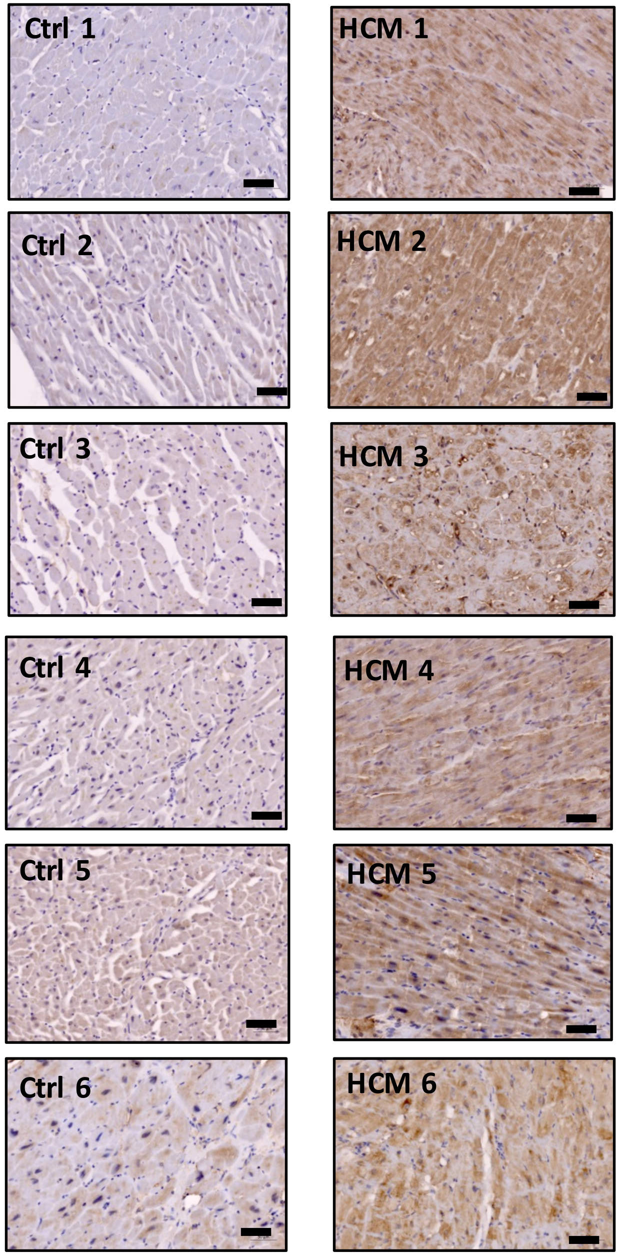

Supplementary Figure 1: FBLN2 elevation in HCM tissues

Representative immunohistochemistry images from control (n=6) and HCM tissues (n=6) show the elevation of FBLN2 in HCM tissues in the cardiomyocytes and the interstitial stroma. Scale bars are 50 µm

Supplementary Figure 2

A

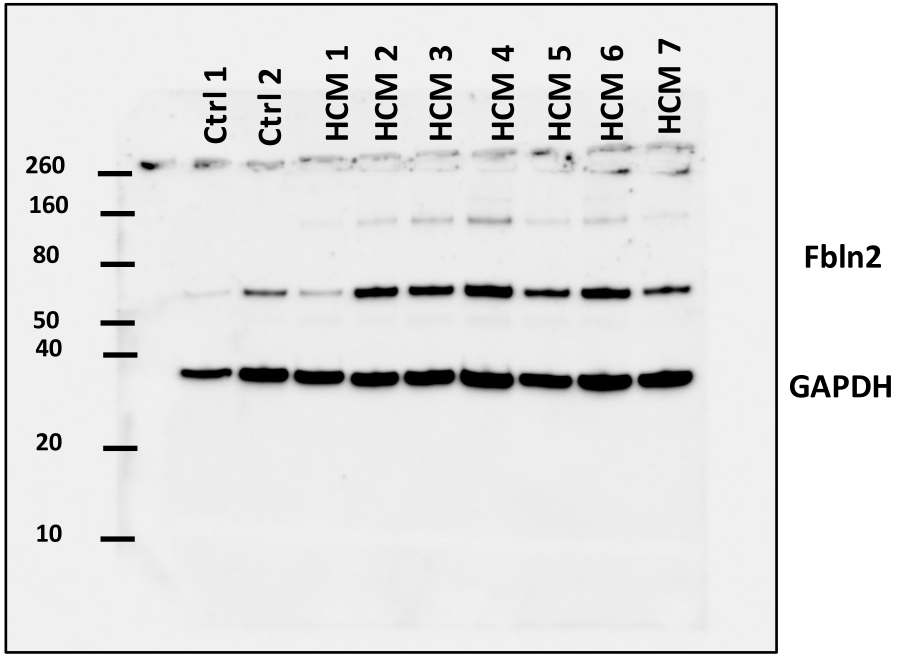

B

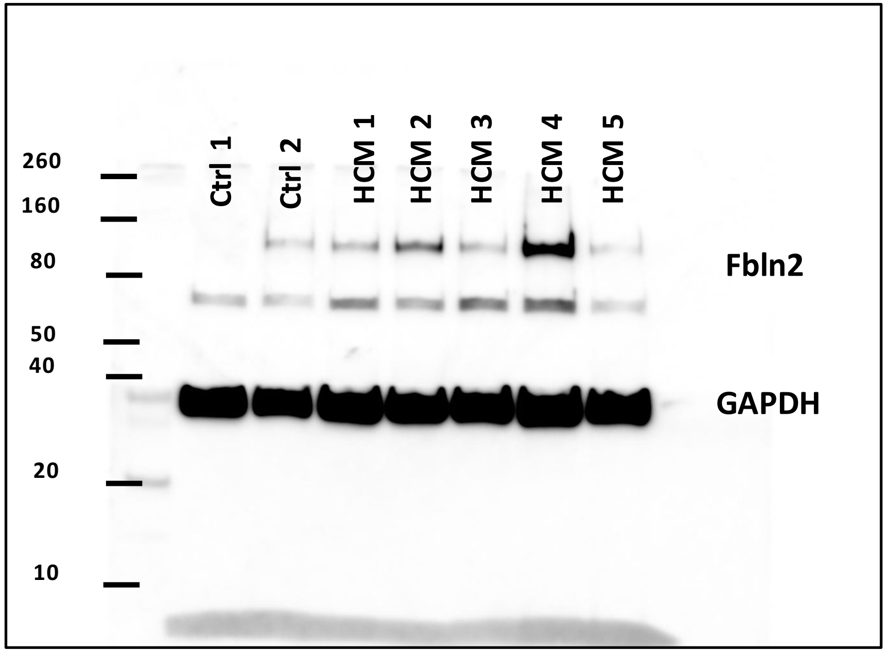

Supplementary Figure 2: [Original images for blots exhibited in Figure 3]

A: A blot for 2 control vs 7 HCM tissues, and B: a blot for 2 control and 5 HCM-associated fibroblasts. Each blot show FBLN2 and GAPDH bands, in addition to the standard protein ladder.

Supplementary Figure 3

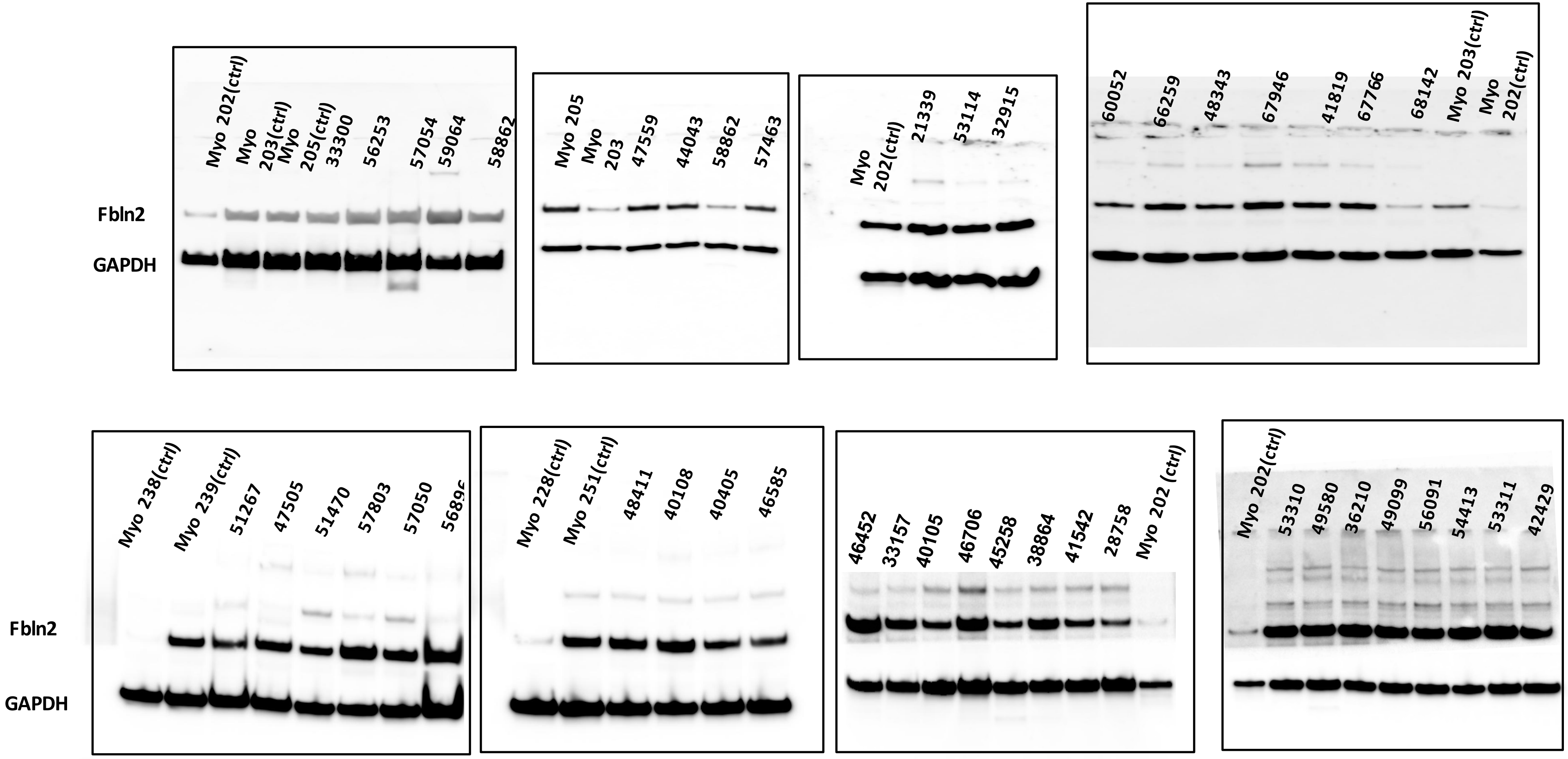

Supplementary Figure 3: FBLN2 protein expression in HCM tissues.: Immuno-blots for HCM (n=44) and controls tissues (n=7; labelled in the blots) show FBLN2 protein expression normalized to GAPDH. All blots used for protein quantification are presented in Figure 3B.

Supplementary Figure 4

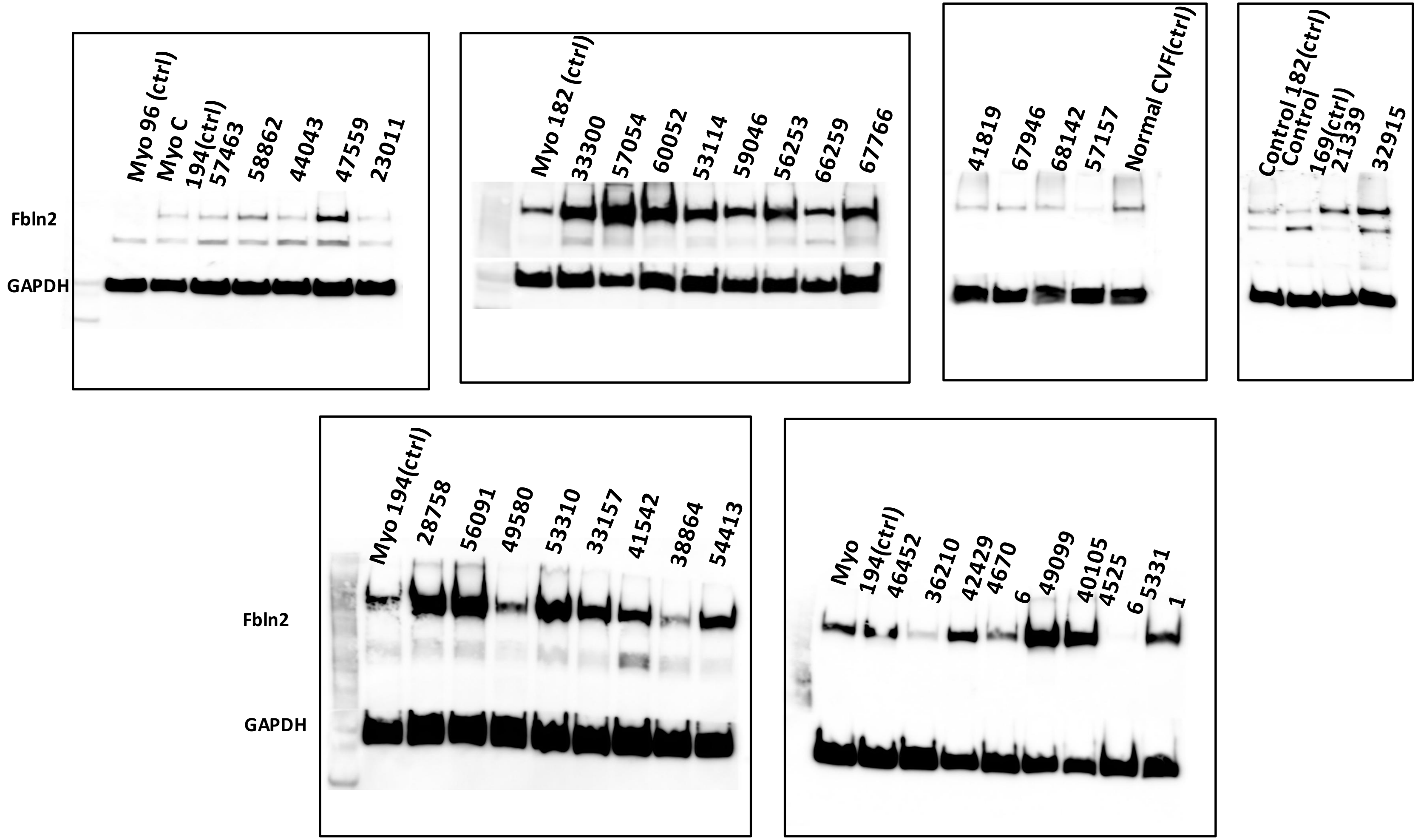

Supplementary Figure 4: FBLN2 protein expression in HCM-derived cultured fibroblasts.
Immuno-blots of cultured fibroblasts derived from HCM (n=34) and controls (n=5; labelled in the blots) show FBLN2 protein expression normalized to GAPDH. All blots used for protein quantification are presented in Figure 3D.
